# Supplementary material for: Structural Heterogeneity of Proteoform-Ligand Complexes in Adenosine Monophosphate-Activated Protein Kinase Uncovered by Integrated Top-Down Mass Spectrometry
Source: J Am Chem Soc. 2025 Aug 14;147(34):30809–19. doi: 10.1021/jacs.5c06950 (PMC12367072; doi:10.1021/jacs.5c06950)
Supplement: Supplementary file 1 [file ja5c06950_si_001.pdf]

Supporting Information

Structural Heterogeneity of Proteoform-Ligand Complexes in Adenosine Monophosphate-Activated Protein Kinase Uncovered by Integrated Top-Down Mass Spectrometry

Hsin-Ju Chan[1], Boris Krichel[2,3,5,6], Liam J. Bandura[1], Emily A. Chapman[1], Holden T. Rogers[1], Matthew S. Fischer[1], David S. Roberts[1], Zhan Gao[2], Man-Di Wang[1], Jingshing Wu[7], Charlotte Uetrecht[3,5,6], Song Jin[1], Ying Ge*[1,2,4]

[1]Department of Chemistry, University of Wisconsin-Madison, Madison, Wisconsin 53706, USA.

[2]Department of Cell and Regenerative Biology, University of Wisconsin-Madison, Madison, Wisconsin 53705, USA.

[3]School of Life Sciences, University of Siegen, 57076 Siegen, Germany.

[4]Human Proteomics Program, School of Medicine and Public Health, University of Wisconsin-Madison, Madison, Wisconsin 53705, USA.

[5]CSSB Centre for Structural Systems Biology, Deutsches Elektronen-Synchrotron DESY & Leibniz Institute of Virology (LIV) & University of Lübeck, Notkestraße 85, 22607 Hamburg, Germany.

[6]Institute of Chemistry and Metabolomics, University of Lübeck, Ratzeburger Allee 160, 23562 Lübeck, Germany.

[7]Department of Pediatrics, Cardiovascular Research Center, University of Wisconsin-Madison, Madison, Wisconsin, 53705, USA.

*Correspondence should be addressed to Y.G. (ying.ge@wisc.edu)

Table of Contents

Supporting Tables 3

Table S1. AMPK proteoform-ligand complexes identified in native TDMS analysis 3

Table S2. Dissociated AMPK subunit proteoforms and ligand binding identified in complex-up analysis 4

Table S3. AMPK β and γ subunit proteoforms identified in complex-up analysis using in-source collisionally activated dissociation (IS-CAD)..............5

Table S4. AMPK fragments identified in complex-down analysis..............6

Supporting Figures **7**

Figure S1. SDS-PAGE analysis of AMPK affinity purification..............7

Figure S2. Comparison of phosphorylation levels across different ligand binding states 8

Figure S3. Representative mass spectrum of complex-up analysis using in-source collisionally activated dissociation 9

Figure S4. Comparison of AMP binding among dissociated subunits 10

Figure S5. IS-CAD and complex-down analysis of AMPK β subunit..............11

Figure S6. IS-CAD and complex-down analysis of AMPK γ subunit..............12

Figure S7. Native top-down fragmentation map of AMPK α subunit 13

Figure S8. Native top-down fragmentation map of AMPK β subunit 14

Figure S9. Representative AMPK structure annotated with native top-down ECD fragmentation sites..............15

Figure S10. Denatured TDMS analysis of AMPK subunits using online RPLC-Q-TOF MS...16

Figure S11. Denatured TDMS analysis of AMPK α subunit using RPLC-Q-TOF MS 17

Figure S12. Proteoform stoichiometry of AMPK β subunit 18

Figure S13. Denatured TDMS analysis of AMPK γ subunit using FTICR-MS/MS 19

Supporting Tables

Table S1. AMPK proteoform-ligand complexes identified in native TDMS analysis. Experimental most abundant mass (mean ± standard deviation, n = 3), calculated most abundant mass, and mass error for the identified complex. Abbreviations: methionine (Met); phosphorylation (P, phospho); adenosine monophosphate (AMP).

Proteoform-Ligand Complex	Experimental Mass (Da)	Calculated Mass (Da)	ΔMass (Da)	Annotation	
				PTMs	Ligands
AMPK αβγ	153713 ± 1	153714	<1	Met removal*3	
AMPK αβγ + P	153793 ± 4	153794	<1	Met removal*3, phospho*1	
AMPK αβγ + AMP	154060 ± 4	154061	1	Met removal*3	AMP*1
AMPK αβγ + AMP + P	154139 ± 3	154141	1	Met removal, phospho*1	AMP*1
AMPK αβγ + 2 AMP	154407 ± 2	154408	<1	Met removal*3	AMP*2
AMPK αβγ + 2 AMP + P	154486 ± 3	154488	2	Met removal, phospho*1	AMP*2

Table S2. Dissociated AMPK subunit proteoforms and ligand binding identified in complex-up analysis. Experimental most abundant mass, calculated most abundant mass, and mass error for the identified complex. Abbreviations: methionine (Met); phosphorylation (P, phospho); adenosine monophosphate (AMP).

Subunits	Experimental Mass (Da)	Calculated Mass (Da)	ΔMass (Da)	Annotation	
				PTM	Ligand
AMPK β	22341.0	22341.4	0.5	Met removal	
AMPK β + P	22421.2	22421.4	0.2	Met removal, phospho	
AMPK γ	34659.9	34658.5	1.4	Met removel	
AMPK αγ	131376.2	131372.1	4.1	α: Met removal<br>γ: Met removal	AMP*1
AMPK αγ + AMP	131720.6	131719.2	1.4	α: Met removal<br>γ: Met removal	AMP*1
AMPK αγ + 2 AMP	132079.3	132066.2	13.1	α: Met removal<br>γ: Met removal	AMP*2
AMPK αβ	119052.5	119055.1	2.6	α: Met removal<br>β: Met removal	
AMPK αβ + P	119132.4	119135.0	2.6	α: Met removal<br>β: Met removal, phospho	
AMPK αβ + AMP	119406.6	119402.1	4.5	α: Met removal<br>β: Met removal	AMP*1
AMPK αβ + AMP + P	119488.4	119482.1	6.3	α: Met removal<br>β: Met removal, phospho	AMP*1

Table S3. AMPK β and γ subunit proteoforms identified in complex-up analysis using in-source collisionally activated dissociation (IS-CAD). Experimental most abundant mass, calculated most abundant mass, and mass error for the identified complex. Abbreviations: methionine (Met); phosphorylation (P, phospho); gluconoylation (G, glucono); phosphogluconoylation (PG, phosphoglucono); adenosine monophosphate (AMP).

Subunit Precursor	Experimental Mass (Da)	Calculated Mass (Da)	ΔMass (Da)	Error (ppm)	Annotation	
					PTM	Ligand
AMPK β (12+)	22341.6	22341.4	0.1	5.8	Met removal	
AMPK β + P (12+)	22421.6	22421.4	0.2	6.8	Met removal, phospho	
AMPK γ (16+)	34658.8	34658.5	0.3	8.5	Met removal	
AMPK γ + G (16+)	34835.9	34835.5	0.3	9.7	Met removal, glucono	
AMPK γ + PG (16+)	34915.8	34915.5	0.3	8.4	Met removal, phosphoglucono	
AMPK γ + AMP (16+)	35005.9	35005.5	0.3	9.1	Met removal	AMP

Table S4. AMPK fragments identified in complex-down analysis. For MS/MS spectra of complex-down analysis in **Figure S4** and **Figure S5**, fragment ion type, charge, experimental monoisotopic mass, calculated monoisotopic mass, and mass error were listed.

Subunit (CE)	Ion	Charge	Exp Mass (Da)	Calc Mass (Da)	Error (ppm)
β, 12+ (CE=40V)	y42	3	4910.67	4910.67	-0.6
	y42	2	4910.66	4910.67	2.1
	b27	2	3034.55	3034.54	-1.9
	b32	2	3598.94	3598.94	0.0
	b36	2	4052.10	4052.10	-0.3
	b41	3	4595.44	4595.44	0.1
	b42	3	4710.47	4710.47	-0.5
	b42	2	4710.47	4710.47	0.5
	b43	3	4823.55	4823.56	0.3
	b47	3	5235.75	5235.71	-6.4
	b55	3	6300.22	6300.22	0.0
	b61	3	7022.58	7022.54	-5.6
	b61	4	7022.53	7022.54	1.4
γ, 16+ (CE=50V)	y20	1	1981.18	1981.18	1.1
	y23	1	2339.29	2339.29	0.9
	y33	1	3456.92	3456.92	-1.0
	y41	2	4395.50	4395.47	-6.4
	y53	2	5781.16	5781.18	2.3
	y68	3	7564.09	7564.09	0.0
	y74	3	8284.39	8284.40	1.4
	y82	3	9122.84	9122.89	6.0
	y88	3	9876.27	9876.26	-1.0
	y142	6	15831.39	15831.40	1.1
	b18	1	2076.88	2076.88	1.1
	b20	1	2303.04	2303.05	1.5
	b30	1	3376.60	3376.62	6.4
	b30	2	3376.62	3376.62	0.1
	b34	2	3805.85	3805.84	-1.6
	b35	2	3904.90	3904.91	3.5
	b54	3	5990.05	5990.07	2.8
	b102	5	11846.06	11846.10	4.0
	b105	6	12208.32	12208.30	-1.7
	b114	6	13102.85	13102.76	-6.3
γ , 16+ (CE=60V)	y20	1	1981.18	1981.18	3.0
	y23	1	2339.29	2339.29	0.2
	y33	1	3456.93	3456.92	-2.4
	y68	3	7564.10	7564.09	-0.6
	y88	3	9876.15	9876.26	11.4
	b18	1	2076.88	2076.88	1.0
	b19	1	2189.95	2189.96	4.2
	b20	1	2303.04	2303.05	1.6
	b30	1	3376.62	3376.62	0.2

Supporting Figures

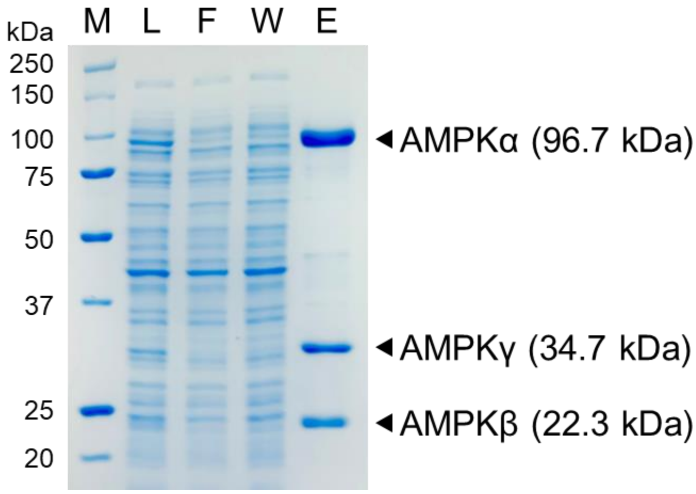

Figure S1. SDS-PAGE analysis of AMPK affinity purification. AMPK was purified using a maltose binding protein tag. Samples loaded in each lane were labeled. M: marker, L: lysate, F: flow through, W: wash, E: elution.

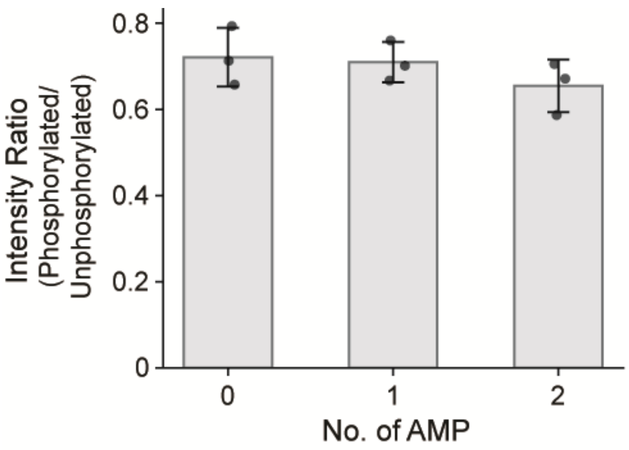

Figure S2. Comparison of phosphorylation levels across different ligand binding states. The intensity ratio of phosphorylated and unphosphorylated AMPK when binding to 0, 1, or 2 AMP molecules. Data are presented as mean ± standard deviation (n = 3).

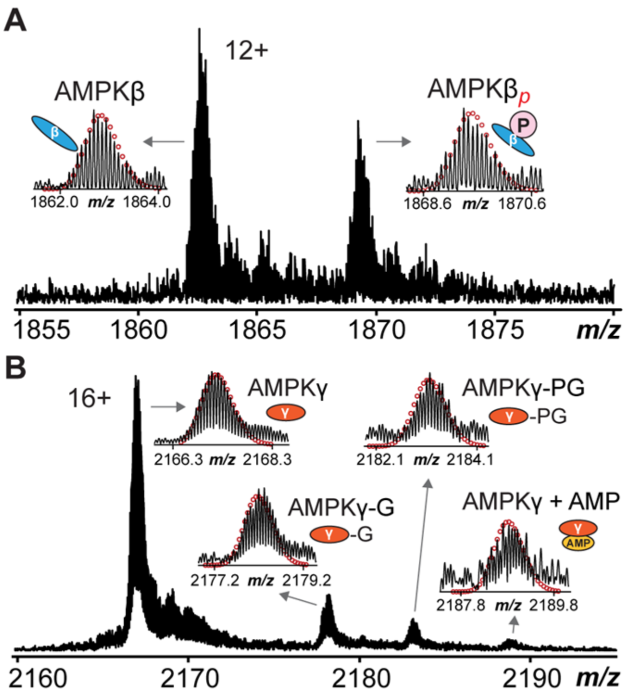

Figure S3. Representative mass spectrum of complex-up analysis using in-source collisionally activated dissociation. The mass spectrum was acquired with an acquisition size of 2M. Zoomed-in views of (A) AMPK β (z = 12+) and (B) AMPK γ (z = 16+). P: phosphorylation, G: gluconoylation, PG: phosphogluconoylation, AMP: adenosine monophosphate. Theoretical isotopic distributions are indicated by the red circles.

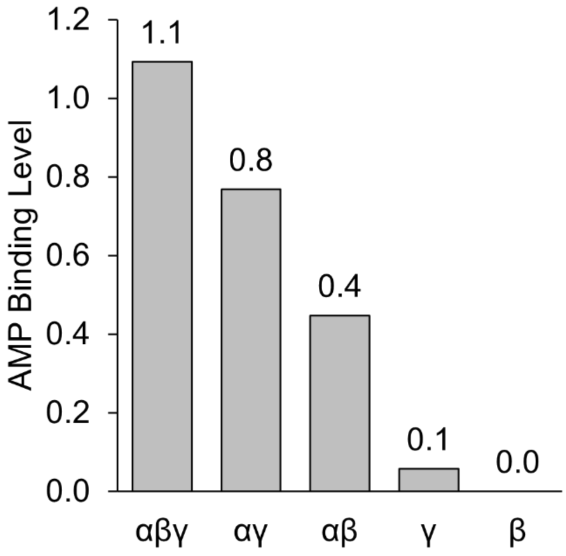

Figure S4. Comparison of AMP binding levels among dissociated AMPK subunits and the intact trimeric complex. AMP binding levels were calculated using the following equation:

$$\text{AMP binding level} = \frac{(I_0 \times 0) + (I_1 \times 1) + (I_2 \times 2)}{I_0 + I_1 + I_2}$$

where I_0 is the intensity of the unbound species, I_1 is the intensity of the singly bound species, and I_2 is the intensity of the doubly bound species.

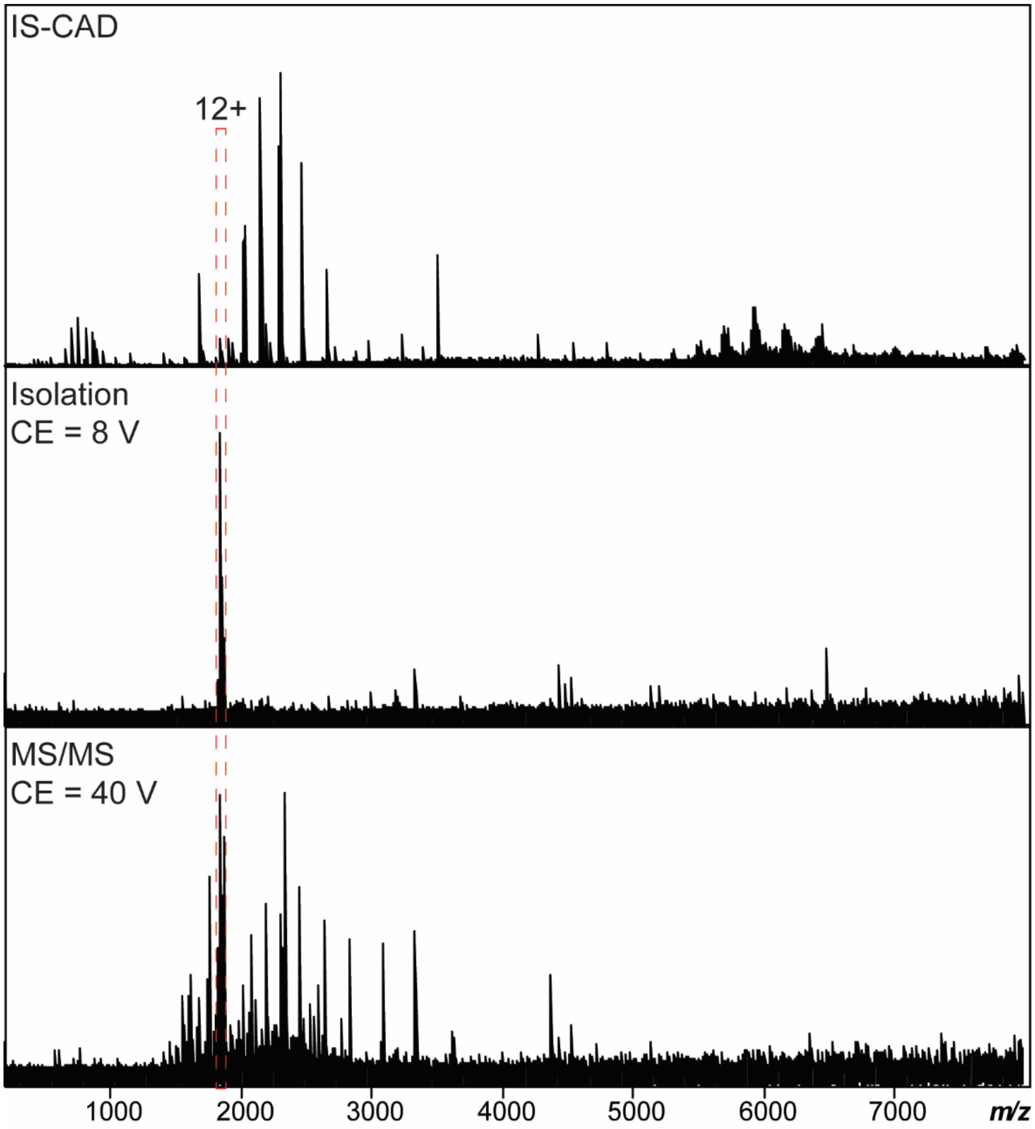

Figure S5. IS-CAD and complex-down analysis of AMPK β subunit. MS/MS characterization of the isolated β subunit (z = 12+). Subunit dissociation was induced by applying IS-CAD in the funnel skimmer region (funnel 1 = 180 V and skimmer 1 = 160 V). For precursor isolation and complex-down MS/MS, a CAD energy of 8 V and 40 V were applied in the collision cell, respectively.

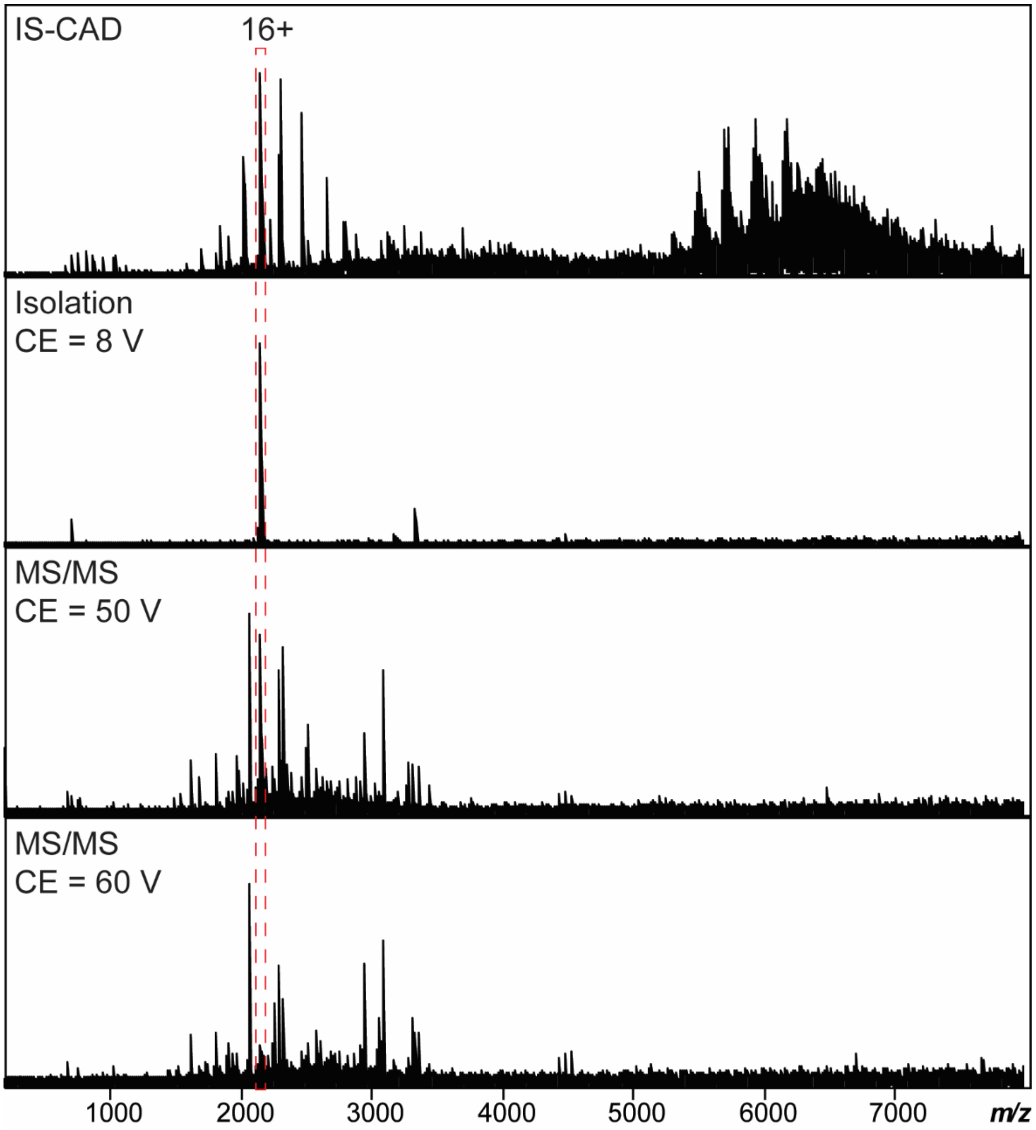

Figure S6. IS-CAD and complex-down analysis of AMPK γ subunit. MS/MS characterization of the isolated AMPK γ subunit (z = 16+). Subunit dissociation was induced by applying IS-CAD in the funnel skimmer region (funnel 1 = 180 V and skimmer 1 = 170 V). For precursor isolation and complex-down MS/MS, a CAD energy of 8 V, 50 V, and 60 V were applied in the collision cell, respectively.

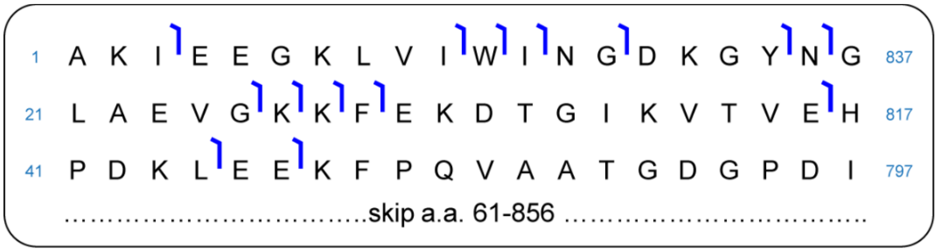

Figure S7. Native top-down fragmentation map of AMPK α subunit. Intact AMPK complex was fragmented using electron-capture dissociation (ECD) resulting in sequence informative *c* ions confirmed within a 20-ppm mass error tolerance. The sequence table shows the characterization of AMPK α subunit. The native top-down ECD data for AMPK α generated 14 *c* ions achieving 1.6% total bond cleavage.

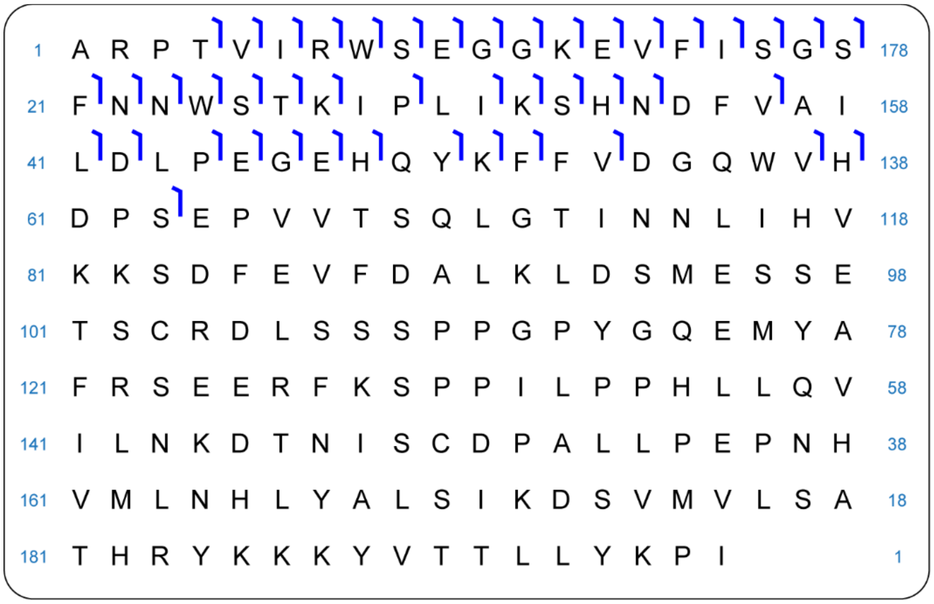

Figure S8. Native top-down fragmentation map of AMPK β subunit. Intact AMPK complex was fragmented using ECD resulting in sequence informative *c* ions confirmed within a 20-ppm mass error tolerance. The sequence table shows the characterization of AMPK β subunit. The native top-down ECD data for AMPK β generated 45 *c* ions achieving 23% total bond cleavage.

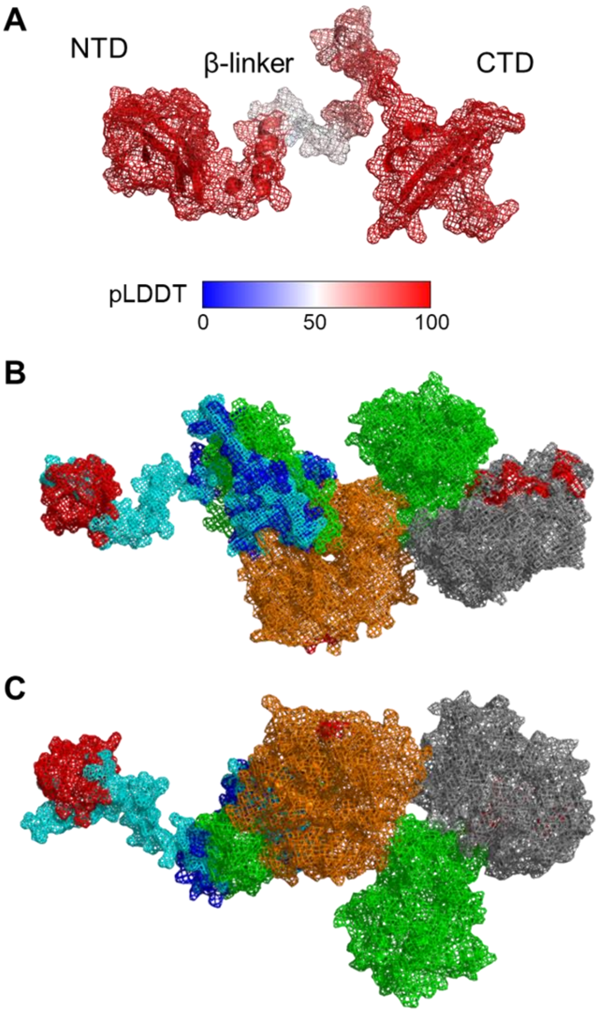

Figure S9. Representative AMPK structure annotated with native top-down ECD fragmentation sites. (A) AlphaFold predicted β subunit (AF-O43741-F1-v4) colored by predicted local distance difference test (pLDDT) confidence values. NTD: N-terminal domain; CTD: C-terminal domain. AMPK heterotrimeric complex (PDB: 7M74) was aligned and merged with full-length β subunit predicted by AlphaFold (AF-O43741-F1-v4). (B) Front view. (C) Back view. The experimental structure of α, β, and γ is labeled in green, dark blue, and orange, respectively. The maltose binding protein tag fused to the N-terminus of the α is labeled in gray. The predicted structure of the β subunit is labeled in cyan. Bond cleavage sites are labeled in red.

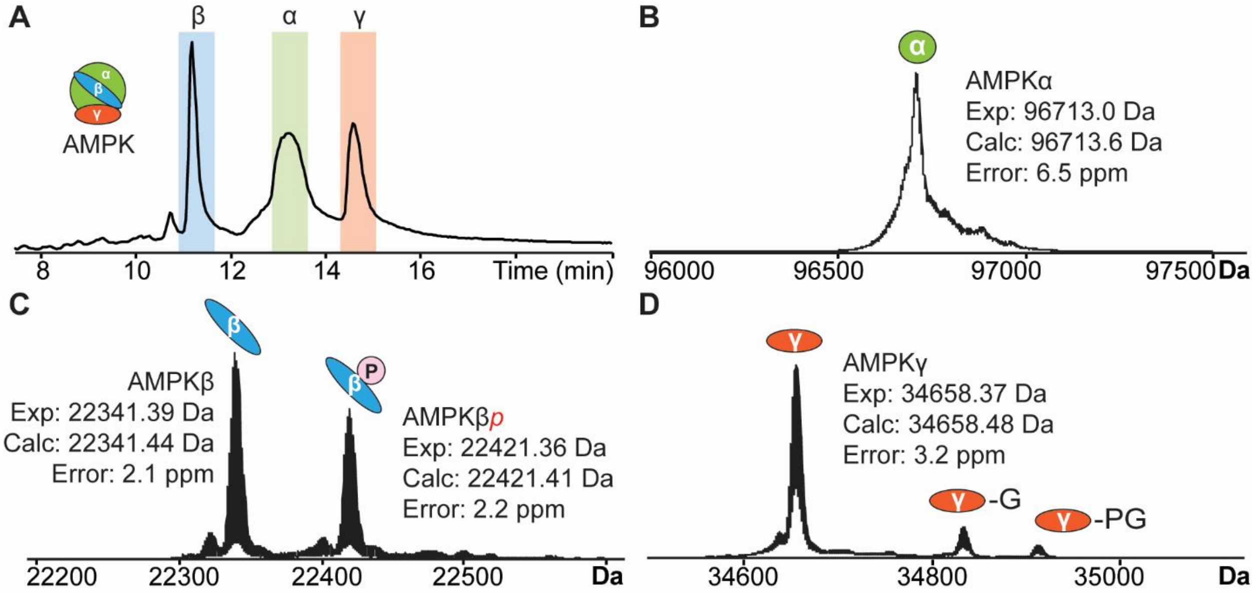

Figure S10. Denatured TDMS analysis of AMPK subunits using online RPLC-Q-TOF MS. (A) Total ion chromatogram of AMPK. The elution windows of the 3 subunits are labeled. Deconvoluted spectra of (B) α, (C) β, and (D) γ subunits. Experimental masses, calculated masses, and mass errors were reported.

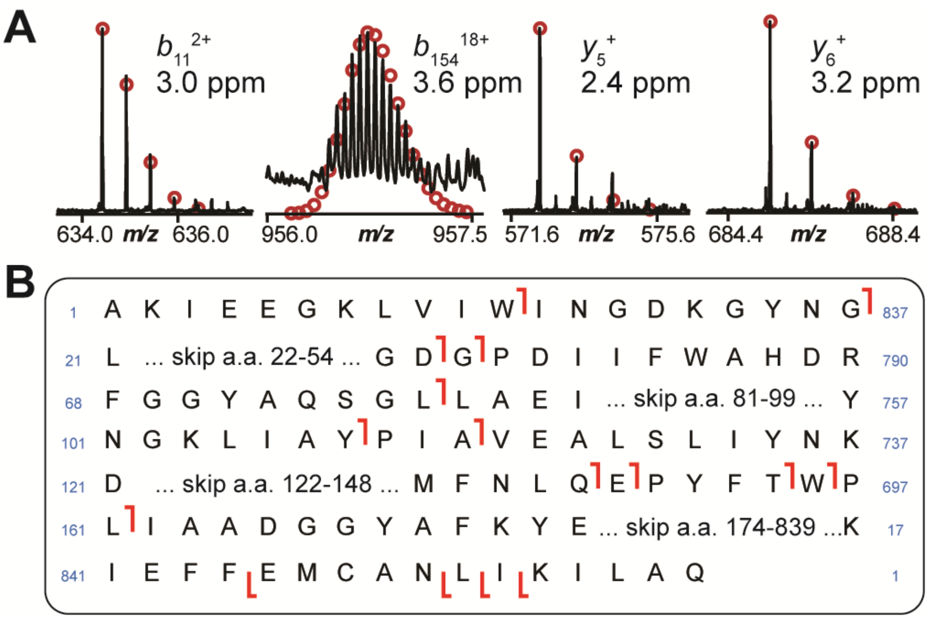

Figure S11. Denatured TDMS analysis of AMPK α subunit using RPLC-Q-TOF MS. (A) Representative CAD fragment ions (b_{11}^{2+}, b_{154}^{18+}, y_5^+, y_6^+) from denatured TDMS analysis. The isotopic fitting is shown with red circles and mass errors are reported. (B) Sequence map of the α subunit annotated with identified CAD fragments.

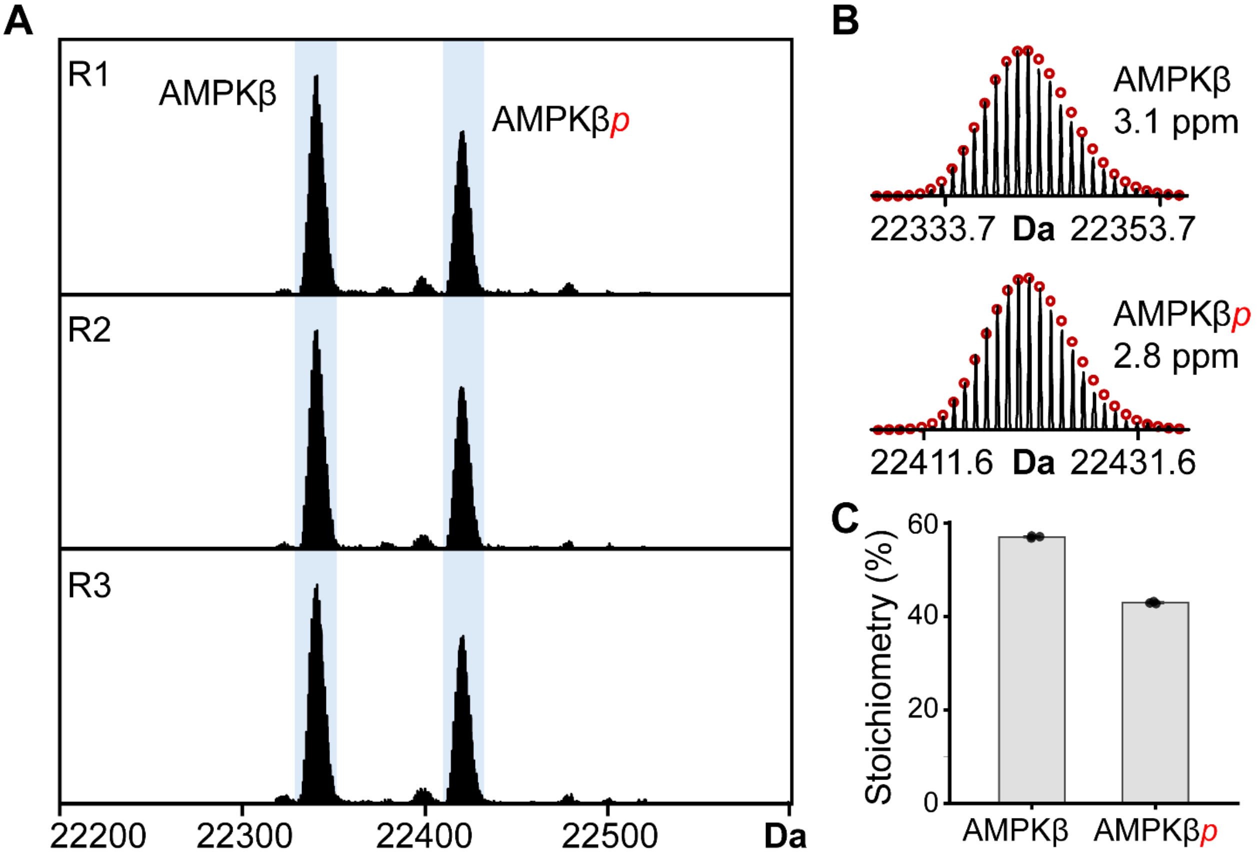

Figure S12. Proteoform stoichiometry of AMPK β subunit. (A) Representative deconvoluted mass spectra of AMPK β proteoforms (R1-3: technical triplicates). AMPKβ: unphosphorylated, AMPKβ*p*: monophosphorylated. (B) Isotope distribution of identified AMPK β proteoforms overlaid with the theoretical isotopic fitting (red circles). Mass errors are reported. (C) Proteoform stoichiometry of unphosphorylated and monophosphorylated AMPK β subunit. Data are presented as mean ± standard deviation (n = 3).

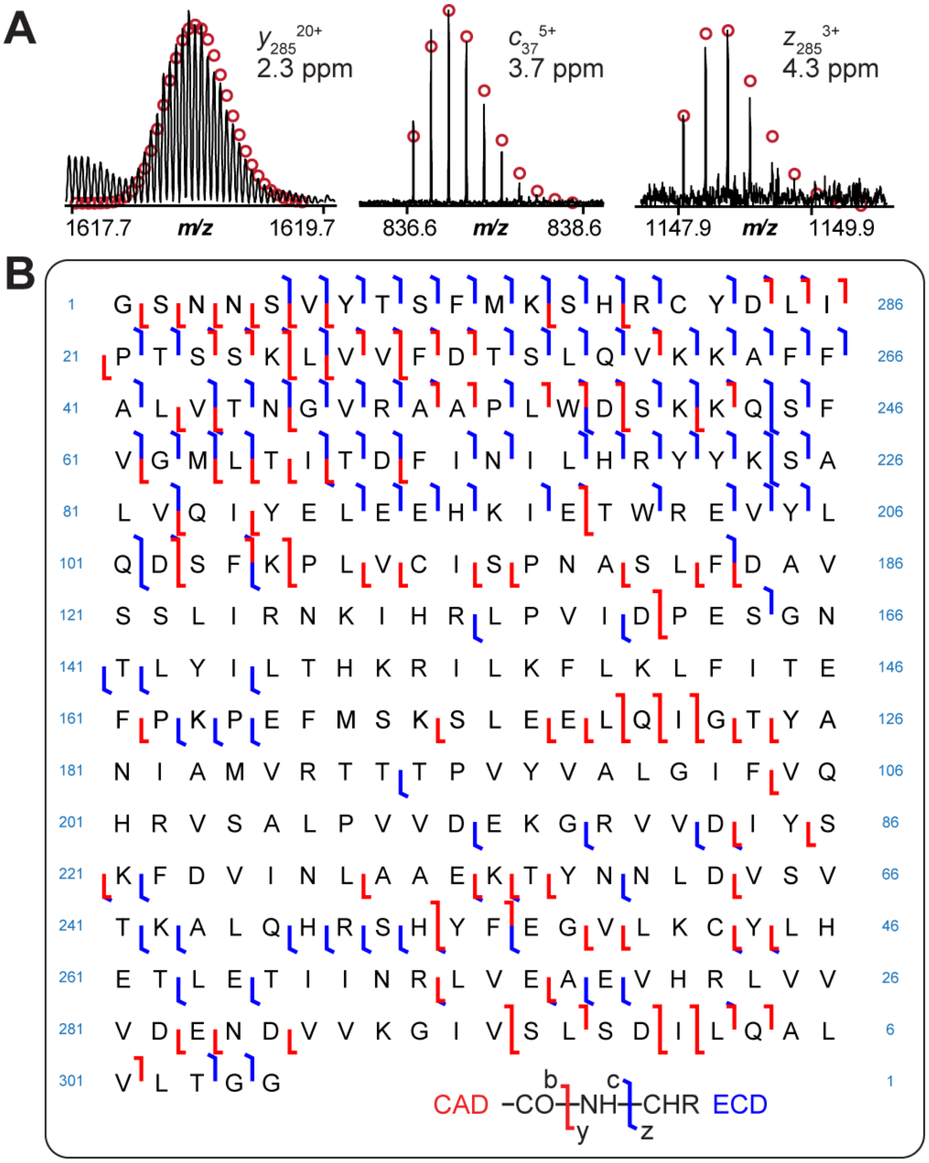

Figure S13. Denatured TDMS analysis of AMPK γ subunit using FTICR-MS/MS. (A) Representative CAD and ECD fragment ions (b_{285}^{20+}, c_{37}^{5+}, z_{33}^{3+}) from denatured TDMS analysis. The isotopic fittings are shown in red circles and mass errors are reported. (B) Sequence map of the γ subunit annotated with identified CAD and ECD fragments.
